# Clinicoetiological Profile of Acute Respiratory Distress Syndrome and Its Association With the Sequential Organ Failure Assessment Score and Clinical Outcomes in a Tertiary Care ICU in Central India: A Prospective Observational Cohort Study

**DOI:** 10.7759/cureus.111451

**Published:** 2026-06-24

**Authors:** Raghuvanshi Ayush Atul, Shilpa Kuthe, Tejas Patil, Shivani Solanki, Shyamal Parve, Anshul Patel, Gandhi Pooja Pramod, Anaya Kamalapurkar, Aniket Hanumant Kothawale, Rohit Rathod

**Affiliations:** 1 Department of General Medicine, N.K.P. Salve Institute of Medical Sciences and Research Centre and Lata Mangeshkar Hospital, Nagpur, IND; 2 Department of General Medicine, Acharya Vinoba Bhave Rural Hospital (AVBRH), Delhi, IND

**Keywords:** acute respiratory distress syndrome (ards), central india region, clinicoetiological, icu patients, mortality, pulmonary critical care, sequential organ failure assessment (sofa)

## Abstract

Background: Acute respiratory distress syndrome (ARDS) is a serious condition frequently encountered in intensive care units (ICUs) and is associated with high morbidity and mortality. Regional data are crucial for optimizing diagnosis and management, particularly in infection-prevalent regions like Central India.

Objectives: This study aimed to evaluate the clinicoetiological profile of ARDS patients and assess the prognostic value of Sequential Organ Failure Assessment (SOFA) scores at admission and 48 hours.

Methods: This prospective observational cohort study included 100 adults who met the Berlin definition of ARDS and were admitted to a tertiary care ICU between January 2023 and January 2025. Demographic data, etiologies, comorbidities, arterial blood gas parameters, SOFA scores, ICU stay, and outcomes were analyzed, and p < 0.05 was considered significant.

Results: The patient population consisted of individuals aged 20-80 years, with a mean age of 54.8 ± 12.3. Notably, 62% were male patients. Community-acquired pneumonia (25%) and sepsis (20%) were leading causes, followed by aspiration pneumonitis (17%). Common comorbidities included hypertension (28%), diabetes (22%), and chronic obstructive pulmonary disease (19%). At admission, patients were classified according to the Berlin Definition into mild, moderate, and severe ARDS based on the partial pressure of oxygen in arterial blood (PaO₂)/fraction of inspired oxygen (FiO₂) ratio. The mean ICU stay was 7.8 ± 4.2 days, and overall mortality was 60%. Significant associations were observed between ARDS severity and clinical outcome (p = 0.001); between SOFA scores at admission and at 48 hours and mortality (p < 0.0001); and between SOFA score and length of ICU stay (p < 0.0001). Mortality increased progressively with increasing ARDS severity and higher SOFA scores.

Conclusions: Infection-related ARDS, predominantly due to pneumonia and sepsis, was the major contributor to ICU mortality. Elevated SOFA scores and low PaO₂/FiO₂ ratios were strong predictors of poor outcome. Early recognition and serial organ dysfunction monitoring are essential to improve survival.

## Introduction

Acute respiratory distress syndrome (ARDS) is a life-threatening form of acute respiratory failure characterized by diffuse alveolar damage, severe hypoxemia, and reduced lung compliance, occurring in the absence of left atrial hypertension. Despite advances in critical care medicine, ARDS continues to impose a substantial global burden, with high morbidity, mortality, and resource utilization in intensive care units (ICUs).

ARDS has undergone several definitional refinements, culminating in the Berlin definition in 2012, which standardized diagnostic criteria based on timing, imaging findings, origin of edema, and severity of hypoxemia [1]. More recently, the 2023 Global Definition of ARDS was introduced to address limitations in the Berlin criteria, particularly by expanding the scope of eligible patients, such as those receiving high-flow nasal cannula or noninvasive ventilation, and incorporating parameters like oxygen saturation (peripheral capillary oxygen saturation/fraction of inspired oxygen (FiO₂)) to improve global applicability, especially in resource-limited settings [2]. These refinements continue to stratify ARDS into mild, moderate, and severe categories based on oxygenation impairment, which remains central to its prognostic implications.

Pathophysiologically, ARDS represents a diffuse inflammatory injury to the alveolar-capillary membrane, resulting in increased vascular permeability, protein-rich pulmonary edema, and surfactant dysfunction [3]. The disease evolves through three overlapping phases, exudative, proliferative, and fibrotic, each contributing distinctly to impaired gas exchange. This pathophysiological process has been well described in earlier landmark studies.

The exudative phase, occurring within the first one to seven days, is characterized by diffuse alveolar damage, endothelial and epithelial injury, and accumulation of protein-rich fluid within the alveolar spaces. This leads to alveolar collapse, decreased lung compliance, and severe ventilation-perfusion mismatch, resulting in refractory hypoxemia.

The proliferative phase typically begins after the first week and is marked by the proliferation of type II pneumocytes and fibroblasts, and the partial restoration of alveolar integrity. Although some degree of lung recovery occurs during this phase, persistent inflammation and interstitial edema continue to impair oxygenation.

In the fibrotic phase, which may develop in a subset of patients, there is extensive fibrosis and remodeling of lung architecture, leading to irreversible loss of functional alveolar units, increased dead space ventilation, and prolonged respiratory failure. These progressive structural and functional changes collectively contribute to impaired gas exchange and are responsible for the high morbidity and mortality associated with ARDS. Understanding these pathophysiological mechanisms is essential for guiding ventilatory strategies and predicting disease progression in patients with ARDS.

The global incidence of ARDS varies widely, ranging from 10 to 80 cases per 100,000 person-years, influenced by population demographics, regional infection patterns, and healthcare infrastructure. In developing countries, infectious etiologies such as pneumonia, sepsis, and aspiration pneumonitis predominate, whereas trauma and transfusion-related lung injury are more common in developed nations. Moreover, approximately one-third of patients initially presenting with mild ARDS progress to moderate or severe disease, underscoring the importance of early recognition and intervention. Similar findings have also been reported in multicenter cohort studies from Asia.

Epidemiological studies such as the Large observational study to UNderstand the Global impact of Severe Acute respiratory FailurE (LUNG SAFE) study have demonstrated that ARDS affects approximately 10% of ICU patients globally and is associated with mortality rates ranging from 30% in mild cases to over 45%-50% in severe cases [4]. The incidence and etiological profile of ARDS vary widely across regions. In developing countries, infectious causes such as pneumonia, sepsis, and tropical infections predominate, whereas trauma and transfusion-related lung injury are more common in developed settings.

Early identification of patients at high risk of poor outcomes is critical in guiding management strategies. Among various prognostic tools, the Sequential Organ Failure Assessment (SOFA) score has emerged as a reliable indicator of organ dysfunction and predictor of mortality in critically ill patients [5]. Serial assessment of the SOFA score, particularly within the first 48 hours, has been shown to correlate strongly with outcomes and may provide dynamic insights into disease progression. Similarly, the partial pressure of oxygen in arterial blood (PaO₂)/FiO₂ ratio remains a key parameter for assessing disease severity and predicting prognosis in ARDS.

Although advances such as lung-protective ventilation strategies, including low-tidal-volume ventilation demonstrated by the Acute Respiratory Distress Syndrome Network trial, have improved outcomes, mortality remains unacceptably high, particularly in resource-limited settings [6]. Factors such as delayed presentation, high burden of infectious diseases, limited ICU resources, and comorbid conditions further complicate management in these regions.

Given the heterogeneity in etiologies, clinical profiles, and healthcare infrastructure, region-specific studies are essential to better understand the patterns of ARDS and to identify locally relevant prognostic factors. Data from Central India remain limited, particularly regarding the role of serial SOFA scoring and its relationship with outcomes.

Therefore, the present study was conducted to evaluate the clinicoetiological profile of ARDS patients admitted to a tertiary care ICU in Central India and to assess the prognostic significance of SOFA scores at admission and after 48 hours. Insights from this study may aid in early risk stratification, optimize management strategies, and improve clinical outcomes in similar healthcare settings.

## Materials and methods

Study design

This was a prospective observational cohort study conducted in the Department of General Medicine at the N.K.P. Salve Institute of Medical Sciences and Research Centre and Lata Mangeshkar Hospital, a tertiary care teaching hospital located in Nagpur, India, and affiliated with the Maharashtra University of Health Sciences, Nashik.

Study duration

The study was conducted between January 2023 and January 2025.

Sample size

The sample size was calculated using the standard formula for estimating a proportion in descriptive studies:

$n = \frac{Z^2 \times p \times (1-p)}{d^2}$

where n is the required sample size, Z is the standard normal deviate at a 95% confidence level (1.96), p is the expected proportion of outcome (mortality in ARDS) based on previous studies (assumed to be 50% due to variability in reported literature), and d is the allowable error (10%). Based on this calculation, the minimum sample size was approximately 96, which was rounded to 100 patients for convenience and completeness.

Inclusion criteria

Patients aged 18 years and older, diagnosed with ARDS according to the Berlin Definition (2012), who were admitted to the medical ICU during the study period, and for whom informed consent was obtained from either the patient or their next of kin, were included in the study.

Exclusion criteria

Patients with postoperative or trauma-related ARDS referred from other centers were excluded.

Participants

Adult patients (≥18 years) who met the Berlin definition of ARDS were enrolled using a consecutive sampling technique during the study period. Inclusion required acute onset within seven days of a known clinical insult, bilateral opacities not fully explained by effusion or nodules, respiratory failure not fully explained by cardiac failure or fluid overload, and PaO₂/FiO₂ ratio ≤300 mmHg with positive end-expiratory pressure (PEEP) ≥5 cm H₂O, as defined by the Berlin criteria [1]. All eligible patients admitted to the medical ICU during the study period who met the inclusion criteria were included consecutively until the desired sample size was achieved. This approach minimized selection bias and ensured representation of the real-world clinical spectrum of ARDS in the study setting.

SOFA score

The SOFA score was used to quantify organ dysfunction. The SOFA score evaluates six organ systems (respiratory, cardiovascular, hepatic, coagulation, renal, and neurological), with each component scored from 0 to 4, and higher scores indicating greater severity of organ dysfunction. The score was calculated at admission and at 48 hours for all patients as described by Vincent et al. [5].

Use of clinical scores

The SOFA score is a widely used clinical tool for assessment of organ dysfunction in critically ill patients. It is not a copyrighted or proprietary instrument and is freely available for use in clinical research. The scoring system was applied in accordance with its original description by Vincent et al. [5].

Data collection

Demographic details, presenting features, comorbidities, etiological workup (including microbiology, serology, and imaging), arterial blood gas parameters, ventilatory settings when applicable, SOFA scores at admission and at 48 hours, length of ICU stay, and outcome at discharge were recorded on a structured proforma. Laboratory investigations included complete blood count, renal and liver function tests, serum electrolytes, procalcitonin, where available, blood and respiratory cultures, and specific tests for tropical infections (such as scrub typhus IgM enzyme-linked immunosorbent assay, dengue serology, and malaria antigen testing) as clinically indicated.

Outcome measures

The primary outcome measure was in-hospital mortality during ICU admission. Secondary outcome measures included length of ICU stay, the association between admission and 48-hour SOFA scores and mortality, and the association between the admission PaO₂/FiO₂ ratio and clinical outcomes. Clinical outcome was categorized as survival or mortality at the time of ICU discharge.

Statistical analysis

Statistical analysis was performed using Statistical Package for the Social Sciences software version 20.0 (IBM Corp., Armonk, NY). Continuous variables were expressed as mean ± standard deviation, while categorical variables were presented as frequencies and percentages. The Student’s t-test was used for comparison of continuous variables between the groups, and the chi-square test or Fisher’s exact test was used for categorical variables as appropriate. A p value of <0.05 was considered statistically significant. Categorical variables were compared using the chi-square test or Fisher’s exact test, as appropriate.

Ethical considerations

Institutional Ethics Committee approval was obtained prior to the commencement of the study. Informed consent was obtained from all participants or their legal surrogates, and patient confidentiality was strictly maintained throughout the study.

## Results

A total of 100 patients with ARDS were enrolled during the study period. The age of participants ranged from 20 to 80 years, with the highest proportion in the 51-60-year age group (30%). Male patients constituted 62% of the study population and females 38% (Table 1).

**Table 1:** Distribution of participants according to age and gender Data are presented as numbers (percentages). No inferential statistical test was applied (descriptive analysis)

Age group (years)	Male, n (%)	Female, n (%)	Total, n (%)
20-30	6 (6%)	3 (3%)	9 (9%)
31-40	9 (9%)	4 (4%)	13 (13%)
41-50	10 (10%)	6 (6%)	16 (16%)
51-60	19 (19%)	11 (11%)	30 (30%)
61-70	11 (11%)	6 (6%)	17 (17%)
71-80	7 (7%)	8 (8%)	15 (15%)
Total	62 (62%)	38 (38%)	100 (100%)

Community-acquired pneumonia was the most common etiology (25%), followed by nonpulmonary sepsis (20%) and aspiration pneumonitis (17%). Tropical infections and healthcare-associated pneumonia each accounted for 9% of cases (Table 2).

**Table 2:** Distribution of participants according to etiology Data are expressed as numbers (percentages)

Sr. no.	Etiological factor	n (%)
1	Community-acquired pneumonia	25 (25%)
2	Healthcare-associated pneumonia	9 (9%)
3	Ventilator-associated pneumonia	5 (5%)
4	Sepsis (nonpulmonary)	20 (20%)
5	Aspiration pneumonitis	17 (17%)
6	Snake bite	2 (2%)
7	Acute pancreatitis	7 (6%)
8	Tropical infection	9 (9%)
9	Connective tissue disorder	3 (3%)
10	Poisoning	3 (3%)
Total		100 (100%)

Hypertension was the most common comorbidity (28%), followed by diabetes mellitus (22%), chronic obstructive pulmonary disease (19%), and ischemic heart disease (17%) (Table 3).

**Table 3:** Distribution of participants according to comorbidity Data are expressed as numbers (percentages) COPD: chronic obstructive pulmonary disease

Sr. no.	Comorbidity	n (%)
1	Diabetes mellitus	22 (22%)
2	COPD	19 (19%)
3	Ischemic heart disease	17 (17%)
4	Chronic kidney disease	9 (9%)
5	Hypertension	28 (28%)
6	Chronic liver disease	6 (6%)
7	None	10 (10%)

Based on the Berlin definition, 30% of patients had severe ARDS (PaO₂/FiO₂ <100 mmHg), 50% had moderate ARDS (PaO₂/FiO₂ 100-200 mmHg), and 20% had mild ARDS (PaO₂/FiO₂ >200 to 300 mmHg) at admission (Table 4).

**Table 4:** Distribution of participants according to PaO2/FiO2 ratio at admission Data are expressed as numbers (percentages) PaO₂: partial pressure of oxygen; FiO₂: fraction of inspired oxygen

Sr. no.	PaO₂/FiO₂ ratio	Frequency
1	<100 (severe)	30 (30%)
2	100-200 (moderate)	50 (50%)
3	>200-300 (mild)	20 (20%)
Total		100 (100%)

Regarding ICU stay, 38% of patients remained in the ICU for one to five days, 36% for 6-10 days, and 26% for more than 10 days (Table 5).

**Table 5:** Distribution of participants according to length of ICU stay (days) Data are expressed as numbers (percentages) ICU: intensive care unit

Sr. no.	Length of ICU stay (days)	n (%)
1	1-5	38 (38%)
2	6-10	36 (36%)
3	>10	26 (26%)

At admission, 38% of patients had mild SOFA scores, 42% moderate SOFA scores, and 20% severe SOFA scores. At 48 hours, among the 95 surviving patients available for reassessment, 25% had mild SOFA scores, 45% moderate SOFA scores, and 25% severe SOFA scores (Table 6).

**Table 6:** Distribution of participants according to SOFA score at admission and after 48 hours Data are expressed as numbers (percentages) SOFA: Sequential Organ Failure Assessment

SOFA category	At admission, n (%)	At 48 hours, n (%)
Mild (0-7)	38 (38%)	25 (25%)
Moderate (8-15)	42 (42%)	45 (45%)
Severe (>16)	20 (20%)	25 (25%)
Total	100 (100%)	95 (95%)

A significant association was observed between ARDS severity and clinical outcome. Mortality increased from 30% in mild ARDS to 64% in moderate ARDS and 73.3% in severe ARDS (χ² = 10.35, p = 0.001) (Table 7).

**Table 7:** Association between PaO2/FiO2 ratio at admission and outcome of patient Chi-square test is applied. Data are expressed as numbers (percentages within outcome group) χ² (1, n = 100) = 10.35, p = 0.001 ARDS: acute respiratory distress syndrome

ARDS severity	Total	Survivors, n (%)	Mortality, n (%)
Mild (>200-300)	20	14 (70%)	6 (30%)
Moderate (100-200)	50	18 (36%)	32 (64%)
Severe (<100)	30	8 (26.67%)	22 (73.33%)
Total	100	40 (40%)	60 (60%)

SOFA score at admission was strongly associated with outcome. Mortality progressively increased with higher SOFA scores and reached 100% among patients with SOFA scores ≥16 (χ² = 66.10, p < 0.0001) (Table 8).

**Table 8:** Association between SOFA score at admission and outcome of the patient Chi-square test is applied. Data are expressed as numbers (percentages within each SOFA category) χ² (4, n = 100) = 66.10, p < 0.0001 SOFA: Sequential Organ Failure Assessment

SOFA range	Survival, n (%)	Mortality, n (%)
0-5	28 (100%)	0 (0%)
6-10	10 (37%)	17 (63%)
11-15	2 (8%)	23 (92%)
16-20	0 (0%)	10 (100%)
>20	0 (0%)	10 (100%)

Similarly, SOFA score at 48 hours demonstrated a significant association with mortality. Patients with SOFA scores ≥16 at 48 hours had mortality rates approaching 100% (χ² = 67.60, p < 0.0001) (Table 9).

**Table 9:** Association between SOFA score at 48 hours and outcome of the patient Chi-square test is applied. Data are expressed as numbers (percentages within each SOFA category) χ² (4, n = 95) = 67.60, p < 0.0001 SOFA: Sequential Organ Failure Assessment

SOFA range	Survival, n (%)	Mortality, n (%)
0-5	17 (100%)	0 (0%)
6-10	20 (80%)	5 (20%)
11-15	3 (11%)	25 (89%)
16-20	0 (0%)	21 (100%)
>20	0 (0%)	4 (100%)

A significant association was also observed between SOFA category and ICU length of stay (χ² = 27.85, p < 0.0001). Patients with severe SOFA scores were more likely to require prolonged ICU admission exceeding 10 days (Table 10).

**Table 10:** Association between SOFA score and length of ICU stay (days) Chi-square test is applied. Data are expressed as numbers (percentages within the SOFA category) χ² (4, n = 100) = 27.85, p < 0.0001 SOFA: Sequential Organ Failure Assessment; ICU: intensive care unit

SOFA category	1-5 days, n (%)	6-10 days, n (%)	>10 days, n (%)	Total
Mild (0-7)	25 (65.8%)	10 (26.3%)	3 (7.9%)	38
Moderate (8-15)	10 (23.8%)	20 (47.6%)	12 (28.6%)	42
Severe (>16)	3 (15%)	6 (30%)	11 (55%)	20
Total	38 (38%)	36 (36%)	26 (26%)	100

**Figure 1:**
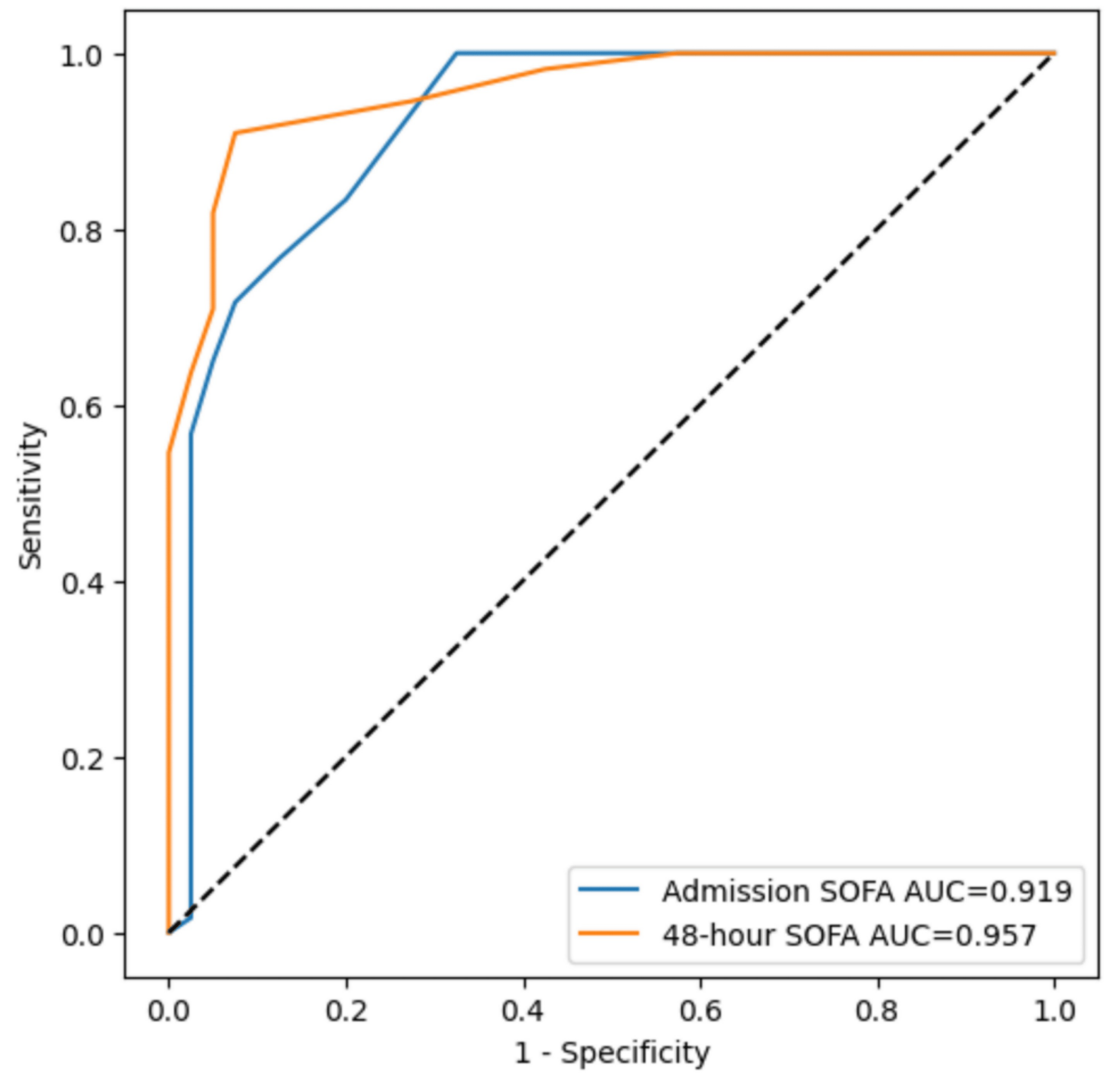
ROC curve analysis for admission and 48-hour SOFA scores in predicting mortality among ARDS patient ROC: receiver operating characteristic; SOFA: Sequential Organ Failure Assessment; ARDS: acute respiratory distress syndrome; AUC: area under the curve

Figure 1 illustrates the relationship between SOFA score at admission and at 48 hours with clinical outcome. Receiver operating characteristic curve analysis was performed to assess the ability of SOFA scores to predict mortality. The admission SOFA score demonstrated excellent discriminatory ability (area under the curve (AUC) = 0.919), whereas the 48-hour SOFA score showed even greater predictive accuracy (AUC = 0.957). The higher AUC for the 48-hour SOFA score suggests that serial assessment of organ dysfunction provides improved prognostic information compared with a single admission measurement.

## Discussion

ARDS remains one of the most complex challenges in critical care due to its varied etiologies, rapid progression, and high mortality despite advances in management. The present study, conducted in a tertiary care hospital in Central India, examined the clinicoetiological profile, comorbidities, SOFA scores, oxygenation indices, and outcomes of ARDS patients, providing region-specific insights comparable to both Indian and international data.

Age and gender distribution

The predominance of middle-aged and elderly patients in this study aligns with global data demonstrating increased susceptibility to ARDS with advancing age [1,3]. Age-related decline in immune response, reduced pulmonary reserve, and higher prevalence of comorbidities contribute significantly to disease severity and poorer outcomes. The observed male predominance is also consistent with prior studies, including the LUNG SAFE cohort [4], and may reflect greater exposure to risk factors such as smoking, occupational hazards, and environmental pollutants.

Etiological factors

In this study, community-acquired pneumonia (25%) and sepsis (20%) were the leading causes of ARDS, consistent with findings from international cohorts, including the LUNG SAFE study [4]. This reinforces the concept that infection remains the primary driver of ARDS globally. However, the presence of tropical infections (9%) and uncommon etiologies such as snakebite and poisoning highlights the distinct regional epidemiology of ARDS in India. While the global burden of ARDS is well documented, data from the Indian subcontinent remain heterogeneous and limited. Previous studies conducted in tertiary care centers in India, such as those by Sharma et al. and Magazine et al., have highlighted the unique challenges posed by high-prevalence infectious etiologies, specifically tropical infections and community-acquired pneumonia, which often present with higher mortality rates compared with cohorts in developed nations [7,8]. The significant association between etiology and oxygenation status (PaO₂/FiO₂ ratio) suggests that direct lung injuries, such as pneumonia, may lead to more severe alveolar damage than indirect causes, consistent with established pathophysiological mechanisms [3].

Previous studies from resource-limited settings have observed that the clinical trajectory of ARDS is frequently complicated by delayed presentation and limited access to advanced supportive therapies such as ECMO [9,10]. Despite these regional insights, there remains a paucity of longitudinal data that specifically examine the dynamic prognostic value of serial SOFA scores in Central Indian patient populations. Our study seeks to bridge this gap by providing a granular assessment of clinicoetiological profiles and by verifying the utility of early, serial assessment of organ dysfunction as a predictor of clinical outcomes in this specific, resource-constrained environment.

Severity assessment and prognostic indicators

The distribution of ARDS severity based on the PaO₂/FiO₂ ratio in this study is comparable to that reported in previous studies. A substantial proportion of patients presented with moderate-to-severe ARDS, reflecting delayed healthcare access and advanced disease at presentation in resource-limited settings.

In the present study, mortality increased progressively with greater ARDS severity, as defined by the Berlin Definition. Patients with severe ARDS (PaO₂/FiO₂ ≤100 mmHg) experienced the highest mortality (73.33%), whereas those with mild ARDS (>200-300 mmHg) had comparatively better outcomes. These findings are consistent with the Berlin Definition and previous international studies demonstrating that worsening hypoxemia is associated with increased mortality and greater organ dysfunction [1]. The observed relationship highlights the importance of early assessment of ARDS severity using standardized PaO₂/FiO₂ categories for risk stratification and prognostication in critically ill patients.

The SOFA score emerged as a strong predictor of both ICU stay and mortality. These findings are in agreement with Seminal research [11] and more recent cohorts [12] demonstrating that the SOFA score is a reliable predictor of mortality in critically ill patients. Patients with higher SOFA scores at admission and worsening scores at 48 hours had significantly poorer outcomes. These findings are in agreement with other studies demonstrating that serial SOFA assessment is a reliable predictor of mortality in critically ill patients [12,13]. The observation that mortality approached 100% in patients with SOFA ≥16 underscores the severity of multiorgan dysfunction in advanced ARDS. Such high mortality thresholds underscore the necessity of early triage and the potential role of prognostic scores in guiding intensive care resource allocation in resource-limited settings. At 48 hours, the SOFA score distribution was calculated among the 95 surviving patients, as five patients died within the first 48 hours.

Recent reviews have highlighted that ARDS is a highly heterogeneous syndrome with distinct clinical and biological subphenotypes, which may explain variations in outcomes despite similar degrees of hypoxemia. Emerging evidence suggests that integrating clinical severity scores, such as the SOFA score, with biological and molecular profiling may improve risk stratification and prognostic accuracy in ARDS [14-16].

Length of ICU stay and SOFA score

The majority of patients (38%) stayed in the ICU for one to five days, while 26% required prolonged stay (>10 days). The significant association between SOFA scores and ICU length of stay is well documented, reflecting the increased intensity of nursing care, specialized monitoring, and therapeutic interventions required for patients with multi-organ dysfunction [5,9]. Patients whose SOFA score worsened or failed to improve after 48 hours had extended ICU courses and higher mortality, consistent with findings from Leonard and Sinha [13], who demonstrated that the change in SOFA score over the first 48 hours is a robust prognostic indicator. For descriptive purposes, SOFA scores were categorized into mild, moderate, and severe groups, while for outcome analysis, finer stratification was used.

Mortality trends and clinical implications

The mortality observed in our study (60%) was higher than that reported in international cohorts, including the LUNG SAFE study [4]. This may be attributable to delayed referral of critically ill patients from peripheral centers, a high burden of infection-related ARDS, greater severity of hypoxemia at presentation, and the predominance of multiorgan dysfunction reflected by elevated SOFA scores. Variations in healthcare infrastructure, availability of advanced supportive therapies, and referral patterns may also contribute to differences in mortality between resource-limited settings and developed healthcare systems [9,10]. Recent literature continues to emphasize that differences in healthcare resources, timing of ICU admission, disease heterogeneity, and access to advanced supportive therapies contribute substantially to variations in ARDS mortality across regions [15,16].

The findings of this study emphasize the importance of early recognition, prompt management of underlying etiologies, and continuous monitoring of organ dysfunction. Serial SOFA scoring provides dynamic assessment and can guide therapeutic decisions, including escalation or de-escalation of care.

Clinical and research implications

From a clinical perspective, this study supports integrating SOFA scoring into routine ICU practice for ARDS patients. Early identification of high-risk patients using the SOFA score and the PaO₂/FiO₂ ratio can facilitate timely interventions such as lung-protective ventilation, prone positioning, and appropriate antimicrobial therapy. From a research standpoint, the study highlights the need for further exploration of region-specific factors, including infectious etiologies and healthcare delivery constraints, that influence ARDS outcomes. The incorporation of biomarkers, genomic profiling, and advanced imaging may further refine prognostic models in the future [14,15,17].

Limitations of the study

This study has several limitations that should be acknowledged. First, it was conducted at a single tertiary care center, which may limit the generalizability of the findings to other healthcare settings with different patient populations and resource availability. Second, the sample size was relatively modest (n = 100), which may reduce the statistical power to detect smaller associations and limit the ability to perform detailed subgroup or multivariate analyses.

Third, although comprehensive clinical and laboratory data were collected, there was no standardized documentation of ventilatory management strategies, including parameters such as tidal volume, plateau pressure, positive end-expiratory pressure, and use of adjunctive therapies like prone positioning or neuromuscular blockade. These factors are known to significantly influence outcomes in ARDS and could act as potential confounders.

Additionally, the study did not assess long-term outcomes, including 28- or 90-day mortality, duration of mechanical ventilation, or functional status and quality of life among survivors. Lastly, because this was an observational study, causal relationships between variables could not be definitively established.

Future directions

Future research should focus on multicenter studies with larger sample sizes to improve the generalizability and statistical robustness of findings. Incorporating standardized ventilatory protocols and treatment strategies would help better evaluate the impact of specific interventions on outcomes.

Longitudinal follow-up of patients to assess long-term mortality, pulmonary function, and quality of life is essential to understand the full spectrum of ARDS outcomes. Additionally, the use of advanced statistical models, including multivariate and survival analyses, may help identify independent predictors of mortality more accurately.

While clinical scores like the SOFA remain essential, future research should integrate biological subphenotyping and biomarker-driven strategies, which have shown promise in identifying "hyper-inflammatory" phenotypes that may respond differently to standard ARDS therapies [18].

Further studies exploring the role of biomarkers, early goal-directed therapies, and region-specific infectious etiologies may provide deeper insights into disease mechanisms and guide personalized management strategies in resource-limited settings.

## Conclusions

This study demonstrates that ARDS in critically ill patients predominantly affects middle-aged and elderly individuals, with a clear male predominance. Community acquired pneumonia was the leading cause, followed by sepsis and aspiration pneumonitis, underscoring the predominance of infection-related etiologies in this population. Hypertension, diabetes mellitus, and chronic obstructive pulmonary disease were the most frequent comorbidities and were significantly associated with prolonged ICU stay and adverse outcomes.

Increasing ARDS severity as defined by the Berlin criteria was strongly associated with adverse outcomes. Patients with severe ARDS (PaO₂/FiO₂ ≤100 mmHg) experienced significantly higher mortality compared with those with moderate and mild ARDS while higher SOFA scores were consistent predictors of poor prognosis. Patients with mild SOFA scores (≤5) showed excellent survival, whereas those with scores ≥11 had progressively worse outcomes, and scores ≥16 were associated with extremely high mortality in this study. The trend of rising SOFA scores at 48 hours further enhanced prognostic accuracy, reflecting the dynamic nature of organ dysfunction.

Overall mortality in this cohort was 60%, emphasizing the gravity of ARDS in resource-limited settings. Age, etiology, comorbidities, PaO₂/FiO₂ ratio, and SOFA score emerged as key determinants of ICU outcomes. Early identification of high-risk patients and serial SOFA monitoring can guide timely interventions, improve prognostication, and potentially reduce mortality. Strengthening infection prevention and optimizing chronic disease management remain crucial for better outcomes in ARDS.
